# Effectiveness of Virtual Reality in Reducing Perceived Pain and Anxiety Among Patients Within a Hospital System: Protocol for a Mixed Methods Study

**DOI:** 10.2196/52649

**Published:** 2024-05-09

**Authors:** Ajay Mittal, Jonathan Wakim, Suhaiba Huq, Tung Wynn

**Affiliations:** 1 College of Medicine University of Florida Gainesville, FL United States; 2 Perelman School of Medicine University of Pennsylvania Philadelphia, PA United States

**Keywords:** virtual reality, digital health, feasibility, acceptability, pain, anxiety, hospital, hospitalization, in-patient, observational study, pharmacologic pain management, pain management, topical anesthetic creams, topical cream

## Abstract

**Background:**

Within hospital systems, diverse subsets of patients are subject to minimally invasive procedures that provide therapeutic relief and necessary health data that are often perceived as anxiogenic or painful. These feelings are particularly relevant to patients experiencing procedures where they are conscious and not sedated or placed under general anesthesia that renders them incapacitated. Pharmacologic pain management and topical anesthetic creams are used to manage these feelings; however, distraction-based methods can provide nonpharmacologic means to modify the painful experience and discomfort often associated with these procedures. Recent studies support distraction as a useful method for reducing anxiety and pain and as a result, improving patient experience. Virtual reality (VR) is an emerging technology that provides an immersive user experience and can operate through a distraction-based method to reduce the negative or painful experience often related to procedures where the patient is conscious. Given the possible short-term and long-term outcomes of poorly managed pain and enduring among patients, health care professionals are challenged to improve patient well-being during medically essential procedures.

**Objective:**

The purpose of this pilot project is to assess the efficacy of using VR as a distraction-based intervention for anxiety or pain management compared to other nonpharmacologic interventions in a variety of hospital settings, specifically in patients undergoing lumbar puncture procedures and bone marrow biopsies at the oncology ward, patients receiving nerve block for a broken bone at an anesthesia or surgical center, patients undergoing a cleaning at a dental clinic, patients conscious during an ablation procedure at a cardiology clinic, and patients awake during a kidney biopsy at a nephrology clinic. This will provide the framework for additional studies in other health care settings.

**Methods:**

In a single visit, patients eligible for the study will complete brief preprocedural and postprocedural questionnaires about their perceived fear, anxiety, and pain levels. During the procedure, research assistants will place a VR headset on the patient and the patient will undergo a VR experience to distract from any pain felt from the procedure. Participants’ vitals, including blood pressure, heart rate, and rate of respiration, will also be recorded before, during, and after the procedure.

**Results:**

The study is already underway, and results support a decrease in perceived pain by 1.00 and a decrease in perceived anxiety by 0.3 compared to the control group (on a 10-point Likert scale). Among the VR intervention group, the average rating for comfort was 4.35 out of 5.

**Conclusions:**

This study will provide greater insight into how patients’ perception of anxiety and pain could potentially be altered. Furthermore, metrics related to the operational efficiency of providing a VR intervention compared to a control will provide insight into the feasibility and integration of such technologies in routine practice.

**International Registered Report Identifier (IRRID):**

DERR1-10.2196/52649

## Introduction

### Overview

Standard pain management protocols in adult medical settings, such as a hospital or clinic, typically rely on the use of pharmacotherapies such as acetaminophen, nonsteroidal anti-inflammatory drugs, and opioids to alleviate acute pain. Although these forms of treatment can be effective, there are growing concerns surrounding the use of opioids and the potential health risks associated with the use of pharmacotherapies under certain conditions. As a result, there is a growing demand to support limiting the use of pharmacotherapy for acute pain in favor of nonpharmacologic options. However, there are few alternative options for providing pain management in adults who often receive inadequate pain control. There is a need to find effective and acceptable alternate forms of pain management and anxiety reduction in a hospital or clinic setting. Virtual reality (VR) could potentially be this alternate form of pain management due to its ability to distract.

Distraction-based therapy techniques are used by hospital staff to help patient cope with injuries, hospitalization, or illness and they differ based on each patient’s needs and preferences. Some common techniques used by specialists include controlled breathing, guided imagery, and relaxation and passive techniques like auditory distraction and television. Studies for each of these different techniques over the years found positive, but mostly mixed results [1]. There is no conclusive study suggesting that 1 technique supersedes others since each patient has different preferences, medical situations, behavioral needs, and developmental needs. Among the adult population, distraction proves beneficial in reducing perceived pain [2]. Distraction became particularly useful for handling illness and procedure-associated pain among adolescents and children with cancer [3,4].

As an emerging technology, VR provides an immersive user experience that shows promise as a tool that can reduce perceived pain and anxiety related to acute pain in an emergency room or other clinical settings through a distraction-based therapy method. Some studies have already used VR technology as a beneficial distractor for pain given its inherent immersive properties [5,6]. With recent advancements, VR technology is being used in numerous settings, but limited research has been done thus far in adult populations.

VR may manage different types of painful experiences, including dressing changes, needle procedures, burns, and postsurgical and chronic pain [7-9]. A study using immersive VR with an adult population suggests that VR can significantly reduce the use of opioids during painful procedures [10].

Successful VR interventions include several factors—the ability to capture attention, enjoyment, immersion, novelty, interactivity, and goal-directedness [11]. Based on the properties of VR, motion sickness and nausea sometimes occur [12]. Therefore, it is necessary to ensure it is safe to use in adults, especially in patients with cancer, by indicating any linked adverse events [13].

There is evidence to support the use of VR as a distraction tool during acute pain events and minor surgeries or procedures. Until recently, the use of VR required a PC, phone, wires, or additional items creating a barrier to adoption in a hospital or clinic setting. The Oculus Quest 2 (Reality Labs) stand-alone headset’s hardware is base priced at US $250, making it an affordable alternative that can potentially provide a nonpharmacologic treatment for acute pain management and anxiety reduction. This study will be using the Oculus Quest 2 as a distraction method because the Quest devices are among the first stand-alone devices commercially available.

The purpose of this pilot study is to evaluate the effectiveness of using nonpharmacological VR distraction methods for specific procedures to reduce perceived anxiety and pain in various medical settings. The information gained will help to design future studies needed to ensure the capability and the reliability of the devices for additional study sites in the future.

### Specific Aims

#### Overview

Specific aims are used to demonstrate the efficacy of the proposed VR intervention in hospital patient populations.

#### Specific Aim 1

Specific aim 1 examines the effect of VR on pain perception before and after a conscious medical procedure in the hospital or outpatient setting.

#### Specific Aim 2

Specific aim 2 examines the effect of VR on perceived anxiety before and after a conscious medical procedure in the hospital or outpatient setting.

#### Specific Aim 3

Specific aim 3 assesses the efficacy of VR as a distraction-based therapy for a conscious medical or dental procedure in the hospital or outpatient setting.

## Methods

### Participants

This research study will use a mixed methods research design to compare the efficacy of using VR as the sole distraction method and the standard of care using other nonpharmacological distraction methods. There will be no alternation to the analgesics or opioids provided to patients during any of their respective medical procedures. Trained research assistants who are familiar with the study protocol for recruitment and eligibility to participate in this research will recruit patients in coordination with medical staff at the respective clinical sites.

The sample population will consist of 250 adults between the ages of 18 and 99 years. The sites this study will operate in include the University of Florida (UF) Health oncology, dental, cardiology, nephrology, and anesthesiology clinics.

Criteria to participate in this study vary between clinical sites based on the conscious medical procedure this study is approved to enroll patients in. Across all sites, this study will only enroll adult (older than 18 years of age) participants. In the oncology ward, adult participants must receive a prescription for a bone marrow biopsy by hospital staff to meet the minimum eligibility requirements. Adult participants from the UF Health Dental clinic coming in for a cleaning will be eligible. Similarly, at the UF Health Cardiology clinic, adult patients undergoing cardiac ablations will be recruited. At the UF Health Nephrology clinic, adult patients undergoing renal biopsies will be recruited. At the UF Health Anesthesiology clinic, adult patients with a broken bone (hand, arm, and leg) receiving an epidural will be recruited.

The study will exclude patients matching the criteria of nausea or vomiting upon admission; require urgent procedures or are otherwise deemed unstable by hospital staff; and have a condition that prevents the use of VR technology such as epilepsy, or a facial or scalp wound. These same exclusion criteria apply to patients enrolled at all UF Health medical or dental clinics.

The VR content displayed to all participants in the experimental group is game developed through an engineering team affiliated with the University of Florida. The VR game requires the user to slightly shift their head to collect tokens and capture images of wildlife going through a nature trail. Current distraction-based methods that are standard in the clinics this study operates in include music.

Participants will be randomly assigned into 1 of the 2 groups. The groups are group 1, participants who received VR as the only distraction method during their medical procedure (n=25 per site; n=125 total), and group 2: participants who received no VR but standard distraction methods during their procedure (n=25 per site; n=125 total). The sites that will be enrolling participants include oncology, dental, cardiology, nephrology, and anesthesiology.

### Recruitment

After identifying eligible participants for this study, trained research assistants will approach and recruit them. If these eligible participants are interested, a trained study member will review and obtain consent directly from the participant. A standardized informed consent script will be adhered to ensure consistency in the consenting process. Participants will receive a copy of the informed consent document.

### Procedure

Research assistants will identify and determine the eligibility of patients admitted in the UF Health Division of Hematology and Oncology wards, UF Dental, UF Cardiology, UF Nephrology, and UF Anesthesiology clinic working in coordination with medical staff. Consent will be obtained directly from the participant.

Before the procedure, medical staff will measure the blood pressure, heart rate, and rate of respiration for participants in both the VR intervention and control groups. These measures are a part of the “standard of care” at all medical or dental clinics in the UF Health system.

All participants, regardless of group, will be asked to complete a procedural anxiety question and to rate their pain using the Verbal Numerical Rating Scale (VNRS) before the procedure. Additionally, patients will be asked to complete the clinically validated Surgical Fear Questionnaire (SFQ) prior to the procedure. Patients will also be asked if they have a history of motion sickness and if they are familiar with VR. A research assistant will assist the patient or participants with putting the VR headset on and turning on a program.

We will begin timing the total length of the procedure as soon as a distraction method begins, that is, the start of a distraction method or when a VR program is turned on, before the procedure. Then, the hospital staff will perform the procedure. During the procedure, vitals (heart rate, respiration rate, and O_2_ stat) will be recorded from a pulse oximeter. Blood pressure will be collected if possible or otherwise recorded via the medical record.

After the procedure is complete, a research assistant will assist patients with removing and shutting off the VR headset and clearing any other distraction method. Timing will stop once the procedure is complete. At the end of the medical procedure, medical staff will remeasure blood pressure, heart rate, and rate of respiration for participants in both groups as part of the standard of care.

Participants, in both groups, will be asked to complete the procedural anxiety question and VNRS post their procedure. Any participant who used VR will be asked to answer questions related to their VR experience via the Likert scale listed in the secondary measures. These questions relate to the perceived effectiveness of the VR intervention and their willingness to use VR in a medical setting in the future. At the end of the study visit, participants will be thanked for their time and participation.

Following the study visit, research assistants will retrieve demographic information (age, sex, race, and ethnicity) on the participants from their electronic medical record. Research assistants will also retrieve the participant’s pre- and postprocedure vitals (blood pressure, heart rate, and rate of respiration) from the participant’s electronic medical record. Data from the site of the procedure will also be collected by research assistants through a retrospective review of the participant’s medical records. This information will be recorded on the preprocedure survey which will be uploaded along with the postprocedure survey digitally to a secured, university-affiliated Dropbox file. Study staff will also sanitize the VR equipment according to the guidelines developed to prevent contamination and the spread of infection.

### Outcome Measures

#### Overview

The following listed measures will be collected for all participants in both groups. All data will be stored via a secured, university-affiliated Dropbox account approved by the institutional review board (IRB), a Health Insurance Portability and Accountability Act (HIPAA) compliant, and university-supported application used for data capture and storage.

#### Primary Measures

##### Pain

The participant will use the VNRS and SFQ to self-report measurement of pain felt before and after the procedure.

##### Verbal Numerical Rating Scale

The VRNS is 1 of the most commonly used validated methods for assessing pain in adult populations [14]. Study staff will ask patients in both groups to rate their pain on a scale of 1 to 10 before the procedure, to establish a baseline pain level, and after the procedure.

##### Time

The time it takes to perform the respective procedures with a nonpharmacologic distraction method is measured. Timing will begin at the beginning of the procedure once the intervention has been set up. Timing will end once the procedure ends.

#### Secondary Measures

##### Anxiety

We will ask the participant a question about their anxiety before and after the procedure using a single anxiety question on a Likert scale.

##### Surgical Fear Questionnaire

The SFQ is a validated 8-question survey that assesses preoperation anxiety and fear [15]. Study staff will ask patients in both groups to rate their fear levels associated with specific questions on a scale from 1 to 10.

##### Procedural Anxiety Question

A 2007 study found that a single question with a Likert scale can quickly and accurately measure anxiety in situations where a full anxiety questionnaire or scale cannot feasibly be used [16]. Study staff will ask patients in both groups about their anxiety level using the question “How worried or anxious are you about the medical procedure?” and the response choices are (1) not anxious or worried at all, (2) slightly anxious or worried, (3) somewhat worried or anxious, (4) very worried or anxious, and (5) extremely worried or anxious.

##### VR Experience

Participants will be asked to rate their VR experience using a 5-point Likert scale on the following statements after being asked the question, “How worried or anxious are you about using VR during this medical procedure?” and the response choices are (1) not anxious or worried at all, (2) slightly anxious or worried, (3) somewhat worried or anxious, (4) very worried or anxious, and (5) extremely worried or anxious. A few open-ended questions regarding their VR experience are “What did you like about using the VR headset?” “What did you not like about using the VR headset?” and “What do you think could be improved about using VR during the procedure?”

##### Demographics and Vitals

Pre- and postprocedure vitals (blood pressure, heart rate, and rate of respiration) and demographic information (age, sex, race, and ethnicity) will be retrieved from the participant’s medical record and stored using the secured, IRB-approved Dropbox.

### Statistical Analyses

Data from the VNRS and procedural anxiety question will be analyzed using paired *t* tests to compare differences between the intervention and control groups. The average time of the suturing procedure will also be compared between groups.

Average ratings for both Likert scales relating to patient experience using VR and hospital staff evaluation of VR use during the procedure will be analyzed for any trends.

### Ethical Considerations

This study, which includes human participant research, was approved by the IRB of the Florida Department of Health (IRB201900850). The informed consent forms, used in this study, provide subjects with a description of the study, its qualitative and quantitative measures, potential risks and discomforts, and explicitly ask the patient to voluntarily agree to participate and allow their data to be collected. Study data are deidentified as all participants are assigned a numerical code. This code follows the format of OC001. Anonymity of all study subjects is ensured. No compensation of any sort is offered to human participants. This is stated in the informed consent form. No identification of individual participants is present in any images of this study or in any supplemental material.

## Results

Our preliminary operations at the oncology site have shown promising results. Anxiety measures indicated a parallel decrease between the control and VR intervention group from preprocedure levels to postprocedure levels with a slightly greater decrease of 0.3 points in reported anxiety in the VR intervention group. Pain measurement comparisons between the control and VR intervention groups indicated a greater difference of decrease in perceived pain among the VR intervention group. The preprocedural pain score for the control group was 2.4 out of 10 and postprocedural pain score was 2.67 out of 10, indicating a 0.27 increase in perceived pain after the procedure was completed. In contrast, the preprocedural pain score for the VR intervention group was 2.95 out of 10 and the postprocedural self-reported pain score was 1.95 out of 10, representing a 1.00 decrease in perceived pain.

Most participants reported enjoyment, comfort, and ease of use when VR was in use. Among the VR intervention group, the average rating for comfort was 4.35 out of 5. A total of 10 respondents mentioned clarity as an area for improvement regarding the visibility of the VR content. Notably, there was an 8.75-minute faster completion of the medical procedure among the VR intervention group (n=125) compared to the control group (n=125).

## Discussion

A total of 50 participants underwent a bone marrow biopsy or lumbar puncture and were randomly assigned to experimental or control groups with 25 VR interventions and 25 control participants included in the analyses. With respect to preliminary findings of clinical effectiveness, results indicated encouraging data trends among the participants in the oncology site with a reported decrease in anxiety and pain and no significant variations in vital sign measurements in the VR intervention group compared to the control group receiving a medical procedure. Thus, the pilot study indicated improvements in the VR group for pain, anxiety, and stress along with quantitative metrics of blood pressure, pulse, and respiratory rate when compared to the control group. Across the entire study period, these differences met the criteria of clinical noninferiority for quality of life. Participants reported feelings of relaxation and enjoyment and reported being highly likely to use the interventions again.

Measurements of feasibility for VR focused on the usability and comfort of the technology, as well as its impact on the length of time for the medical procedure to be completed. Results revealed that the use of VR as a distraction-based relaxation tool in an adult hospital setting is feasible. Participants enjoyed using VR and the majority reported comfort and ease of use when VR was used. Issues related to the clarity of viewing the VR can be addressed through better practices fitting the Oculus Quest 2 headset on the patient. Results also indicated a faster completion of the medical procedure among patients in the VR intervention group. Therefore, the faster completion of the procedure can occur without compromising the quality of care and provides valuable clinical application for VR in the hospital setting as it improves the efficiency of providers while performing routine procedures.

Limitations to the study include the fact that the study was not designed to measure the impact of potential moderators on the outcomes, and the data cannot provide reliable information about the potential impact of age, gender, and pain tolerance of participants enrolled in the study. Additionally, the randomization could not control the use of additional pain medication prior to a medical procedure that was self-prescribed by the patient prior to being approached regarding participation in the study. Furthermore, the study did not factor in medications that the patients are on or the full past medical history of participants to consider the impact of those factors on how the participant’s vital signs report.

Overall, this study indicates that the use of an Oculus Quest 2 Headset with the VR program for both distraction and relaxation is acceptable, feasible, and with preliminary measures, effective for adult patients undergoing a variety of medical procedures by reducing anxiety and pain and reducing procedure time in a hospital setting.

## Abbreviations

HIPAA: Health Insurance Portability and Accountability Act
IRB: institutional review board
SFQ: Surgical Fear Questionnaire
UF: University of Florida
VNRS: Verbal Numerical Rating Scale
VR: virtual reality

## Data Availability

The data sets generated and analyzed during this study are available from the corresponding author on reasonable request.
